# Para-Phenylenediamine Induces Apoptotic Death of Melanoma Cells and Reduces Melanoma Tumour Growth in Mice

**DOI:** 10.1155/2016/3137010

**Published:** 2016-05-17

**Authors:** Debajit Bhowmick, Kaushik Bhar, Sanjaya K. Mallick, Subhadip Das, Nabanita Chatterjee, Tuhin Subhra Sarkar, Rajarshi Chakrabarti, Krishna Das Saha, Anirban Siddhanta

**Affiliations:** ^1^Department of Biochemistry, University of Calcutta, 35 Ballygunge Circular Road, Kolkata 700 019, India; ^2^Cytometry Solutions Pvt Ltd., Tripti Mitra's Garden, Balia Garia Station Road, Kolkata, West Bengal 700084, India; ^3^Cancer & Cell Biology Division, CSIR-Indian Institute of Chemical Biology, 4 Raja SC Mullick Road, Kolkata, West Bengal 700032, India

## Abstract

Melanoma is one of the most aggressive forms of cancer, usually resistant to standard chemotherapeutics. Despite a huge number of clinical trials, any success to find a chemotherapeutic agent that can effectively destroy melanoma is yet to be achieved. Para-phenylenediamine (p-PD) in the hair dyes is reported to purely serve as an external dyeing agent. Very little is known about whether p-PD has any effect on the melanin producing cells. We have demonstrated p-PD mediated apoptotic death of both human and mouse melanoma cells* in vitro*. Mouse melanoma tumour growth was also arrested by the apoptotic activity of intraperitoneal administration of p-PD with almost no side effects. This apoptosis is shown to occur primarily via loss of mitochondrial membrane potential (MMP), generation of reactive oxygen species (ROS), and caspase 8 activation. p-PD mediated apoptosis was also confirmed by the increase in sub-G0/G1 cell number. Thus, our experimental observation suggests that p-PD can be a potential less expensive candidate to be developed as a chemotherapeutic agent for melanoma.

## 1. Introduction

Melanoma is one of the most notorious types of cancer known to us. Studies indicate that melanoma is resistant to most types of chemotherapy by exploiting their intrinsic apoptosis resistance and by reprogramming the proliferative pathways during melanoma development [1–3]. Recent studies show that melanoma cells have the ability to delay apoptotic death [4].

Para-phenylenediamine (p-PD) has long been known to be an essential component of permanent hair dyes [5]. For a long time, p-PD has been used in textile, leather, and hair dye industries [6]. p-PD in the hair dyes is reported to purely serve as an external dyeing agent [5]. Sporadic side effects in human and in mouse model of p-PD are reported [7–10]. Moreover, the experimental designs for some of those reports are not correlative with the real-life use [11–13]. Effects of p-PD on skin and keratinocytes have been thoroughly investigated by Blömeke and her group [14–16]. p-PD is converted to Brandrowski's base by autoxidation and self-conjugation. Besides this, it is shown that keratinocytes do contain N-acetyltransferase 1 (NAT1) which modifies p-PD by mono- and di-N-acetylation, thereby modulating its toxicity [14–16]. The effect of p-PD was investigated in different cell types and was found to cause apoptosis via ROS [17–19]. However, very little is done to demonstrate the consequence of p-PD treatment on melanocytes or melanoma cells.

In this report, we have investigated the effect of p-PD on melanin producing A375 and B16-F10, human and mouse melanoma cell lines, respectively. Here, for the first time, we have demonstrated that p-PD causes disruption of mitochondrial membrane potential and generation of ROS leading to apoptosis of these cells primarily via activation of caspase 8. Our study using mice melanoma model showed that p-PD induced apoptosis to inhibit tumour growth.

## 2. Materials and Methods

### 2.1. Materials

Fetal bovine serum (FBS), Dulbecco's modified Eagle's medium (DMEM), Trypsin-EDTA, and antibiotic and antimycotic agents were obtained from Life Technologies, USA. Monoclonal anti-PARP antibody (9532) and anti-actin antibody (4970) were purchased from Cell Signaling Technology, USA. Anti-caspase 8 (9746) and anti-caspase 9 (9502) antibodies from Cell Signaling Technology, USA, were a generous gift from Dr. Chitra Mondal, Scientist, and anti-JNK antibody (sc-137018), caspase 9 inhibitor (C 1355), caspase 8 inhibitor (C 1230), and PAN caspase inhibitor (C 1355) were from Dr. Pijush. K. Das, Chief Scientist, Indian Institute of Chemical Biology (IICB), Kolkata, India. Anti-Bid antibody (ab77185) from Abcam, USA, was a kind gift from Dr. P. C. Sil from the Department of Molecular Medicine, Bose Institute, Kolkata. Dichlorodihydrofluorescein diacetate (DCFH-DA) is a kind gift from Dr. S. Ghosh from the Department of Biochemistry, University of Calcutta. Alkaline phosphate conjugated secondary antibodies for mouse and rabbit were raised in goat, A3562 and A3687, respectively. MTT based cell counting kit-8 (CCK-8), protease inhibitor cocktail (P2714), N-acetyl cysteine (NAC), p-PD, glutathione reductase, glutathione, NADPH, EDTA, glutathione disulfide (GSSG), DTNB, and all other reagents if not stated otherwise were purchased from Sigma-Aldrich Company, USA. Alexa Fluor® 568 Goat Anti-Rabbit IgG was purchased from Life Technologies, USA.

### 2.2. Cell Line, Culture, and p-PD Treatment

A375 and B16-F10 cells derived from human and mouse melanoma, respectively, used in this paper were purchased from the National Centre for Cell Science, Pune, India. The cell line was cultured in Dulbecco's modified Eagle's media containing 10% fetal bovine serum (Life Technologies, USA) in a humidified atmosphere containing 5% CO_2_ and 95% air at 37°C [20]. The stock solution of p-PD (100 mg/mL) was dissolved in DMSO and different concentrations were prepared in the culture medium with a final DMSO concentration of 0.1%. Both types of cells were seeded at a density of 4 × 10^5^ cells/30 mm plate and grown for 24 h up to 40% confluence. The culture medium was replaced with new medium before adding different concentrations of p-PD to treat the cells for indicated time points. 0.1% DMSO was used as the vehicle control.

For viability assay using MTT, 5000 cells/well were seeded in 96-well plate for 24 hours. Following that, cells were treated with p-PD or 0.1% DMSO as indicated. After the treatment, media in each well were replaced with 100 *μ*L fresh media followed by 10 *μ*L of MTT reagent (CCK-8, Sigma). The absorbance readings were collected at an interval of one hour up to four hours using a Bio-Rad (iMark) microplate reader.

For the studies using caspase inhibitors, the procedure used was very similar to that described above, except that the cells were pretreated with caspase inhibitors for 2 hours and then treated for 24 hours with indicated concentrations of p-PD. In a parallel experiment, MTT assay was done to accurately determine the number of viable cells (absorbance) among cells grown for 24 hours. This value was subtracted from the values obtained from all MTT assays using caspase inhibitors. These corrected values for sets without or with inhibitors were computed using the following equation:

(1) $$\begin{array}{c} \%\text{ change in viable cell}=\left\{\;\left(\;{O_{48}}^{I\text{ or }P}-O_{24}\;\right)\times \frac{100}{O_{24}}\;\right\}, \end{array}$$

where O_48_^I  or  P^ is the absorbance at 48 hours; “I” stands for p-PD treated cells pretreated with caspase inhibitor and “P” stands for cells treated with only p-PD. O_24_ indicates the absorbance after 24 hours of cell seeding. The corrected values for % change in viable cell thus obtained were plotted against respective concentrations of p-PD. The inhibitor concentrations used are as follows: caspase 8 inhibitor (20 *μ*M), caspase 9 inhibitor (15 *μ*M), and PAN caspase inhibitor (40 *μ*M). Cells treated only with these inhibitors do not show any adverse effect.

### 2.3. Immunoblot Analysis

The total cellular protein contents of control and p-PD treated cells were measured and then the cells were boiled in 1x protein loading buffer for 10 minutes. Protein samples were subjected to SDS-PAGE and the gels were transferred onto PVDF membrane. The membrane was blocked in 5% BSA in PBS for 1 h at room temperature with shaking, followed by overnight incubation with respective primary antibodies at 4°C. Alkaline phosphatase conjugated corresponding secondary antibodies in PBS were used for 1 h at room temperature with shaking. At the end of incubation with antibodies, the membranes were washed extensively with PBS containing 0.2% Tween-20. The membranes were developed by the NBT/BCIP system as per the manufacturer's protocol.

### 2.4. Measurement of Glutathione (GSH), Glutathione Disulfide (GSSG), Catalase, and Glutathione Reductase Activity

GSH and GSSG were measured according to the protocols of Akerboom and Sies [21]. Crude cell-free extract was added to an equal volume of 2 M HClO_4_ containing 2 mM EDTA and incubated on ice for 20 minutes. Then, it was centrifuged at 5000 g for 5 minutes. The resulting supernatant was neutralized with 2 M KOH containing 0.3 M HEPES. After centrifugation at 5000 g for 5 minutes, the supernatant was used for estimation of total glutathione (GSH + 2GSSG) by measuring GR-dependent DTNB reduction spectrophotometrically at 412 nm. The same neutralized extract was treated with 2-vinylpyridine (50 : 1, v/v) for 1 hour at room temperature. Then, it was used for GSSG estimation using the above method. Glutathione reductase (GR) activity of crude cell-free extract was determined by measuring NADPH oxidation spectrophotometrically at 340 nm [21]. Catalase activity was measured by the decrease in absorbance at 240 nm spectrophotometrically [22].

### 2.5. Annexin V-Fluorescein Isothiocyanate (FITC)/DAPI Staining Assay

The frequencies of apoptotic cells were detected with Annexin V-FITC (Life Technologies). A375 and B16-F10 cells were incubated with different concentrations of p-PD for various time points. After incubation, cells were trypsinized, collected, washed with PBS, and resuspended in 1x binding buffer (HEPES 10 mM, pH 7.4, 140 mM NaCl, and 2.5 mM CaCl_2_) at a concentration of 1 × 10^6^ cells/mL. In case of tumour cells, they were washed and directly resuspended in binding buffer. Annexin V-FITC and DAPI (1 *μ*g/mL) were added and incubated in the dark for 15 min. Cells were then subjected to flow cytometric analysis on BD FACSVerse (BD Biosciences) using BD FACSuite software. At least 10,000 events were recorded and represented as dot plots. In case of caspase inhibitor studies, we have prepared 3 sets of A375 cells. The first one is A375 control cells, the second is 100 *μ*g/mL p-PD treated cells, and the third is cells that are 100 *μ*g/mL p-PD treated but pretreated with caspase 8 inhibitor. Basal early and late apoptosis values were first subtracted from respective values of 2nd and 3rd group. These modified values are then used to calculate the % of reduction in apoptosis number. Cells positive for only Annexin V-FITC and for both Annexin V-FITC and DAPI were considered as early and late apoptotic cells, respectively.

### 2.6. Flow Cytometric Analysis of Cell Cycle

After p-PD treatment, A375 and B16-F10 cells (1.0 × 10^6^) were harvested and rinsed with PBS. The cell pellets were fixed in 70% ethanol at −20°C overnight. After washing twice with PBS, the cells were incubated with RNase A (1 mg/mL) for 30 minutes at 37°C and then stained using propidium iodide (PI) (50 *μ*g/mL) in PBS, followed by incubation at RT in the dark for 30 min. The samples were analysed by BD FACSVerse (BD Biosciences) using BD FACSuite software. At least 20,000 events were analysed and recorded [23].

### 2.7. Flow Cytometric Analysis of Mitochondrial Membrane Potential

p-PD treated and untreated cells were analysed for their mitochondrial membrane potential (ΔΨm) using JC 1 dye (Life Technologies) according to the manufacturer's instructions. In brief, upon completion of incubation, cells were trypsinized and collected in DMEM. 1.0 × 10^6^ cells were then stained with 2 *μ*M JC 1 at 37°C, 5% CO_2_, for 30 min. Subsequently, the cells were washed and resuspended with PBS prior to flow cytometric analysis in BD FACSAria III (BD Biosciences) using BD FACSDiva software. JC 1 stained normal polarized cells will show high fluorescence in PE channel (PE fluorescence due to the presence of JC 1 aggregates inside the mitochondria) and low fluorescence in FITC channel (FITC fluorescence due to monomeric JC 1 present in the cell); in case of cells containing depolarized mitochondria, the observation will be reversed. At least 10,000 events were analysed and recorded. The data is represented as the percent change in cells with depolarized MMP.

### 2.8. Flow Cytometric Analysis of BrdU Incorporation

We used the BD FITC BrdU Flow Kit for the determination of percentage of cells in S phase following the manufacturer's protocol. In brief, at the end of p-PD treatment, cells were incubated with BrdU for 15 minutes and then they were fixed by BD Cytofix/Cytoperm buffer. After that, cells were treated with BD Cytoperm*™* Permeabilization Buffer Plus Cells were again incubated with BD Cytofix/Cytoperm buffer followed by a DNase treatment to expose BrdU epitopes. Immunofluorescent staining was done using fluorochrome conjugated anti-BrdU antibody. Finally, cells were stained with 7-AAD followed by analysis in BD FACSVerse using BD FACSuite software. At least 20,000 events were recorded and analysed.

### 2.9. Flow Cytometric Analysis of Intracellular ROS Level

Intracellular production of ROS was determined by flow cytometry [24]. Briefly, cells were trypsinized after the p-PD treatment, collected, and washed twice with PBS. 1.0 × 10^6^ cells were loaded with dichlorodihydrofluorescein diacetate (DCFH-DA) (2 *μ*M, Life Technologies) and incubated at 37°C for 15 min in the dark. In case of tumour cells, they were washed and loaded with probe. Then, cells were washed and resuspended in 500 *μ*L of PBS. The samples were analysed by FACSAria III (BD Biosciences) using BD FACSDiva software. At least 10,000 events were analysed and recorded. Viable cells incorporate and deacetylate DCFH-DA to nonfluorescent DCFH which in turn quantitatively oxidized by ROS to produce the fluorescent 2′,7′-dichlorofluorescein (DCF). p-PD treatment shows a positive increase in the median of green fluorescence hence in the quantity of ROS. For ROS depletion studies, A375 cells were pretreated for 1 hour with 5 and 10 mM of glutathione and NAC, respectively, at standard cell growth condition. After 1 hour, p-PD was introduced in the medium for 16 hours. In case of caspase inhibitor studies, pretreatment was done as mentioned earlier.

### 2.10. *In Vivo* Antitumour Activity of p-PD

B16-F10 cells (10^6^ cells/50 *μ*L) were injected subcutaneously (s.c.) on the right flank of Swiss-Albino mice for tumour generation. Care and maintenance of animals were done in adherence to the guidelines of the Institutional Animal Care and Use Committee. The tumour took approximately 2 weeks to become visible. The animals were divided into four groups (*n*, where *n* = 6). One group only contains the mice with no tumour. One tumour bearing mice group was left untreated. The other two groups of tumour bearing mice were given i.p. injections (2 and 4 mg/kg/3 days) of p-PD as per Wilcoxon method [25]. For flow cytometric experiments, single cell suspensions were made from the p-PD treated and untreated mice tumours. For the toxicity study, the animals were divided into three groups (*n* = 10). The first group received vehicle in normal saline i.p. and the second and third groups received p-PD at doses 5 and 10 mg/kg/3 days (dose 1 and dose 2, resp.) i.p. up to 6 weeks. Food and water intake of animals was observed during this period. Twenty-four hours after the last dose on the 44th day, blood was collected from each group by cardiac puncture for estimation of haematological and serum biochemical parameters.

## 3. Results and Discussion

### 3.1. p-PD Mediated Death of Melanoma Cells

To explore the effect of p-PD on melanoma cells, we have treated A375 and B16-F10 cells with different concentrations of p-PD for various time points. Initial investigation under phase contrast microscope showed that the adhered cell number decreases with increasing concentration of p-PD. The time taken for the complete loss of adherent A375 cells was observed to be approximately 20, 2, and 0.5 hours with 1, 10, and 20 mg/mL of p-PD, respectively.

**Figure 1:**
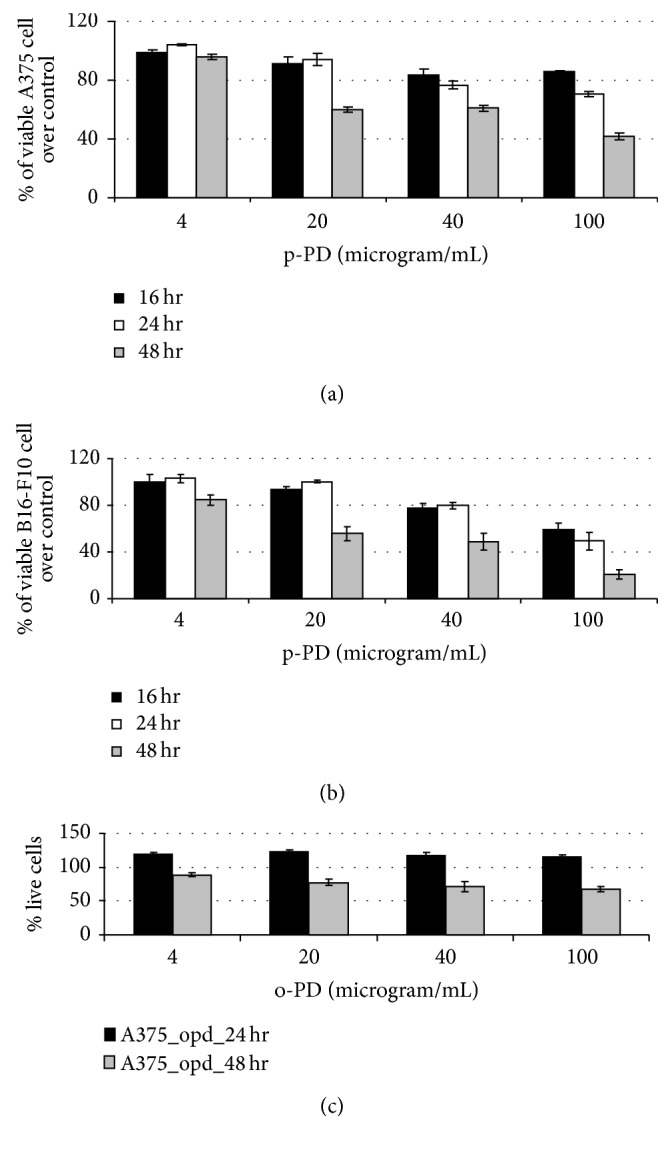
Cytotoxic effect of para-phenylenediamine and ortho-phenylenediamine (o-PD) on melanoma cells. A375 (a) and B16-F10 (b) cells grown in a 96-well (5000 cells in each well) plate were treated with p-PD of indicated concentrations for 16, 24, and 48 hours at 37°C. (c) In a similar manner, A375 cells were also treated with o-PD for 24 and 48 hours with indicated concentrations. Four wells were used for each concentration in every time point. One set of untreated cells (0.1% DMSO) was kept as control for each time point. The absorbance reading (OD) for each set obtained from the microplate reader was used to compute the percent change in viable cell in treated sets over respective controls using the expression {(OD_Control_ − OD_Experimental_) × 100/OD_Control_}. The % viable cell over control was plotted against respective concentrations of p-PD. Error bars represent SD; *n* = 4.

To quantify this cytotoxic effect, we have carried out MTT based cell viability assay using A375 and B16-F10 cells treated with increasing concentrations of p-PD for 6, 16, 24, and 48 hours. At 6 hours' time, p-PD did not show any cytotoxic effect on both cell lines. Figures 1(a) and 1(b) show that about 60% cells remain viable in both cell lines when treated with 20 and 40 *μ*g/mL of p-PD. Treatment with 100 *μ*g/mL of p-PD reduced the viable cells up to 40 and 20% in case of A375 and B16-F10, respectively. When treated with 100 *μ*g/mL of p-PD, B16-F10 cells were found to be more sensitive than A375 at all time points. 0.1% DMSO that was used as the vehicle control showed no effect on both cells.

To investigate whether the cytotoxicity of this compound is specific for the para-isomer, we have carried out similar MTT assay using the melanoma cells treated with different concentrations of o-PD.  Figure 1(c) clearly indicates that while no cell death was observed in all sets treated with similar concentrations of o-PD for 24 hours, treatment for 48 hours had, however, some cytotoxic effect but its extent is much less than that of p-PD.

### 3.2. Intraperitoneal Administration of p-PD Reduced Melanoma Tumour Mass in Swiss-Albino Mice

**Figure 2:**
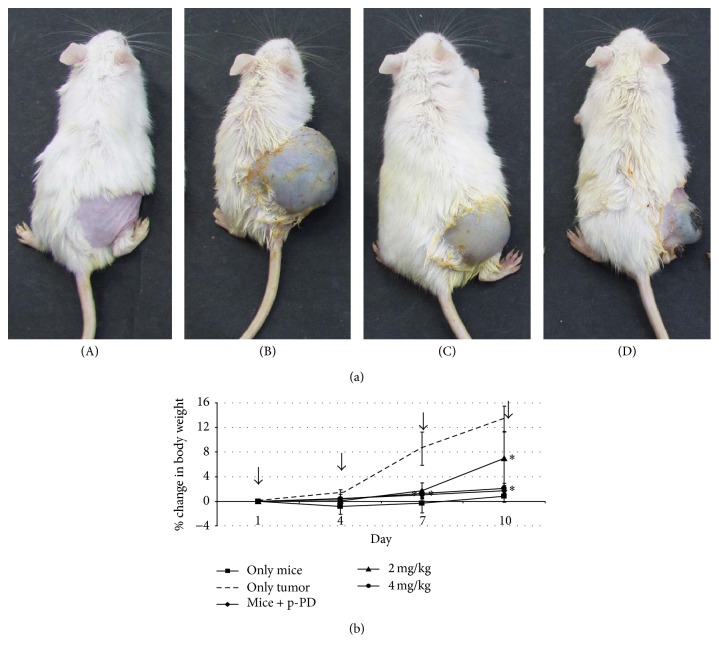
Body weight gain of the mice treated with or without p-PD. (a) Effect of p-PD on tumour development in mice. Initially, right flanks of two sets of Swiss-Albino mice were given subcutaneous injection with only PBS (A) and B16-F10 cells (B) as mentioned in the Materials and Methods. These mice were kept for 2 weeks until visible tumour in the set (B) is formed. The mice from set (B) were divided into three groups: one (C) was treated with 2 and the other (D) was treated with 4 mg/kg of p-PD for 10 days. One (B) set was left untreated as control. Photographs of representative mouse from each set are shown here. Each set comprised 6 mice except for set (B) where initially 18 mice were used for the experiment. (b) Body weights of untreated mice (set (A) above) (■), mice treated with 4 mg/kg of p-PD (⧫), mice having tumour (- - -) (set (B)), and mice having tumour treated with 2 (▲) (set (C)) and 4 (*∙*) mg/kg (set (D)) of p-PD are plotted against the days of treatment. Arrows indicate the time of peritoneal p-PD administration. Error bars represent SD; *n* = 6. *∗* indicates the two-tailed *p* value ≤ 0.018. By conventional criteria, this difference is considered to be statistically significant.

After the determination of the cytotoxic effect of p-PD on melanocytes in culture, we investigated its effect on melanoma tumour in mice. Before that, to assess systemic cytotoxicity of p-PD in mice, two groups of Swiss-Albino mice (male, 3 weeks old) were intraperitoneally (i.p.) injected with p-PD (5 and 10 mg/kg) at an interval of 3 days throughout a period of 6 weeks. After the treatment, the mice appeared to be quite active as the untreated ones (data not shown). Therefore, month-long peritoneal administration of as high as 10 mg/kg of p-PD did not show any sign of toxicity in Swiss-Albino mice. After this, to explore the cytotoxic effect of p-PD on melanoma tumour, we have subcutaneously (s.c.) injected B16-F10 cells (10^6^ cells/50 *μ*L) into the right flank of Swiss-Albino mice for tumour generation. Two weeks later, when the tumours were visible, i.p. injections of 2 and 4 mg/kg of p-PD were given to mice bearing tumours. Our results show that this p-PD treatment to the mice carrying melanoma tumours resulted in reduction of tumour mass as revealed from the tumour size (Figure 2(a); (C) and (D)) and the corresponding decrease in body weight (Figure 2(b)). Cells derived from those p-PD treated tumours showed increased apoptosis as evident from the Annexin V based flow cytometric analysis (data not shown).

There are some reports on toxic effects of p-PD in rodent models. These studies from which the side effects of p-PD were reported are mostly based on unrealistic designs such as feeding [9], topical application [26], and subcutaneous injections [13] of high dosage of p-PD. Besides these, a meagre percentage of the human population is reported to be allergic to p-PD at a concentration of 1% or above [27, 28]. 0.3% p-PD in petrolatum was reported to be reliable with respect to contact dermatitis [26]. Moreover, the concentration range of several standard anticancer drugs which are administered intravenously lies between 1 and 100 mM (FDA approved drugs for oncology) [29]. Maximum concentration of p-PD that we have used is 0.01%, equivalent to a little less than 1 mM. Therefore, according to these published reports, the concentrations of p-PD that we have used are presumed to be very safe and effective in reducing melanoma tumour mass in mice. To further verify this, we have performed haematological and serum biochemical parameters measurement of the treated mice along with the control (up to 6 weeks). Study revealed that there are no mortality and toxic symptoms with no biochemical changes in the major organs of the test groups treated with higher selected dose (10 mg/kg p-PD). No significant differences were found in body weight gain/loss, weight of major organs, and haematological and biochemical parameters of the control and test groups as shown in Tables 1 and 2.

### 3.3. p-PD Induces Apoptosis in Melanoma Cells

**Figure 3:**
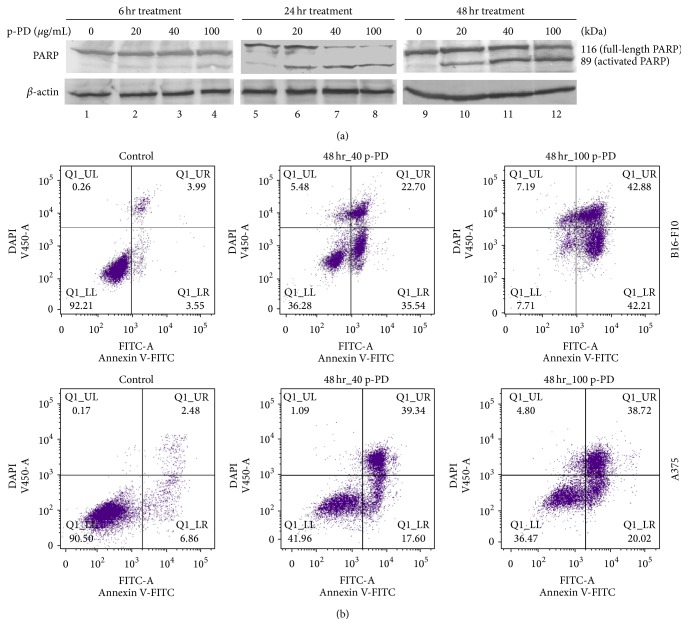
p-PD mediates apoptosis in melanoma cells. (a) A375 cells were treated with p-PD as indicated. After treatment, cells were lysed with protein loading buffer (Materials and Methods). 30 *μ*g of total protein lysate was loaded in each lane and subjected to western blot and probed for activation of PARP. Cells that were not treated with p-PD (0) but with 0.1% DMSO served as control (lanes 1, 5, and 9). These blots are representative of three independent analyses. *β*-actin is used as a loading control for all time points. (b) Both A375 and B16-F10 cells were treated for 48 hours with indicated amounts of p-PD and were subjected to flow cytometry analysis using Annexin V-FITC assay. DAPI was used as the DNA stain.

To understand the mechanism of the cell death by p-PD treatment, we first asked whether it is apoptosis or not. To address that, we have tested the activation of PARP, the well-known marker for apoptosis [30]. To do that, the lysates from A375 cells, treated with indicated concentrations of p-PD at different time points, were analysed by immunoblotting using specific antibody for PARP.  Figure 3(a) shows that the active form of PARP (89 kDa) was present in the treated cell lysates (lanes 2–4, 6–8, and 10–12). The cleaved form of PARP in the untreated samples (lanes 1, 5, and 9) which are much less intense as compared to that of treated samples indicates the presence of naturally occurring apoptotic cell population. It is also clear from this result that either the proportion of the full-length PARP was reduced (compare lanes 6 and 7) or the proportion of the active form of PARP is increased (compare lanes 10 and 11) with the increasing concentration of p-PD treatment.

The apoptosis is also further confirmed by flow cytometry using Annexin V-FITC.  Figure 3(b) shows ~7.5, 58, and 85% of apoptotic B16-F10 cells and ~8.5, 57, and 60% of apoptotic A375 cells when treated with 0, 40, and 100 *μ*g/mL of p-PD, respectively, for 48 hours. The percents of live, early, and late apoptotic cells in control and treated samples are illustrated in Table 3. The table also consists of the number of sub-G0/G1 particles as determined by flow cytometric cell cycle assay. A clear correlation is observed between the apoptotic cell numbers estimated by two different methods. It is to be noted that B16-F10 cells are more sensitive than A375 cells. Moreover, the number of apoptotic cells corroborated with the decrease in live cells as determined by MTT assay (cp. Figures 1(a) and 1(b) with Table 3).

**Figure 4:**
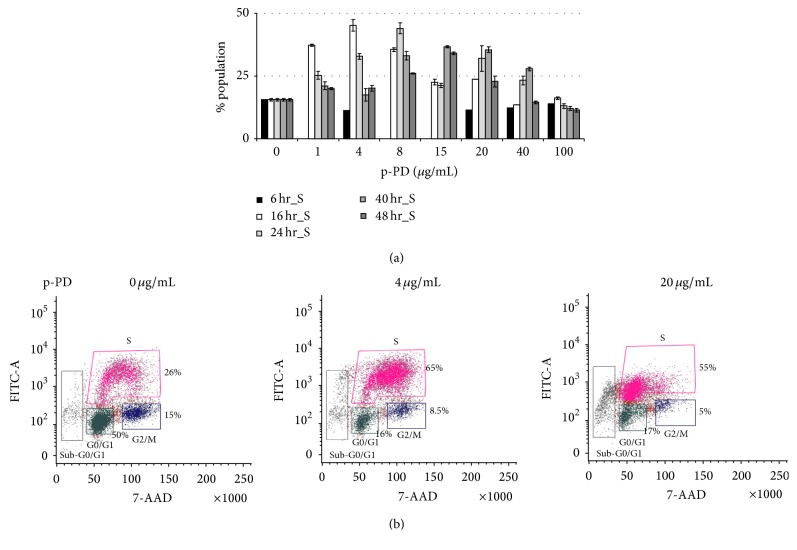
Cell cycle arrest at S phase imposed by p-PD. (a) Cell cycle distribution of A375 cells treated with p-PD as indicated was determined by PI staining using flow cytometry. The number of cells in S phase for each set was determined and plotted against indicated concentration of p-PD. Error bars represent SD; *n* = 3. (b) Flow cytometric analysis of BrdU incorporation by A375 cells treated with indicated concentrations of p-PD. The BrdU positive cell cultures at the S phase are gated in pink. Respective percent cell numbers are shown next to the gated cell clusters representing cell cycle phases.

There are multiple instances where, upon treatment with cytotoxic agents, cells get arrested in a specific cell cycle stage and then undergo apoptosis [31–33]. Therefore, we have also investigated the role of p-PD in cell cycle progression. Our flow cytometric results show that cells specifically treated with low concentrations of p-PD for early time points get arrested in S phase and these blocks go away with time (Figures 4(a) and 4(b)). Treatment with higher concentrations of p-PD was unable to show any such S phase arrest. The reason for this pattern is not clear at this moment. To verify the arrest in the S phase, BrdU incorporation experiment was done using flow cytometry. The data also confirmed the block at S phase in A375 cells treated with low concentration of p-PD (Figure 4(b)). A similar pattern was also found in case of B16-F10 cells (data not shown).

**Figure 5:**
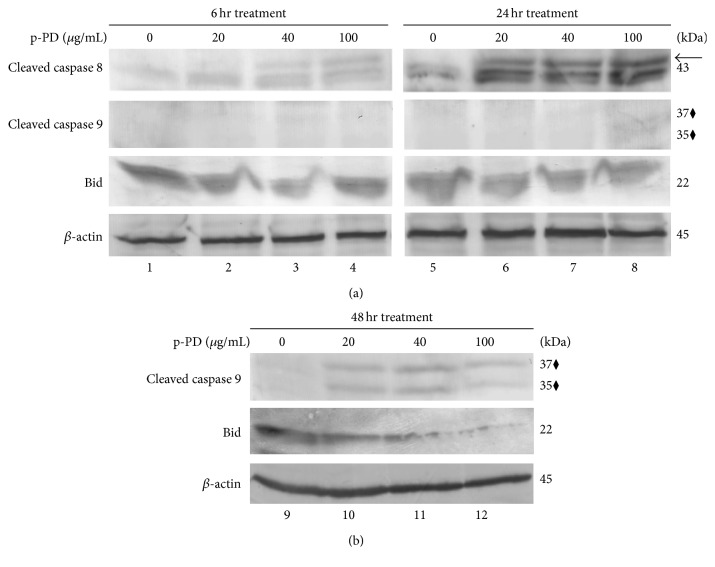
p-PD activates extrinsic apoptotic pathways earlier than the intrinsic ones in human melanoma cells. (a) A375 cells were treated with p-PD as indicated. Cells treated for 6 and 24 hours were lysed with protein loading buffer (Materials and Methods). 30 *μ*g of total protein lysate (same as described in Figure 3(a)) was loaded in each lane and subjected to western blot and probed for activated caspase 3, activated caspase 8, activated caspase 9, and Bid as indicated. Arrow and “⧫” indicate the cleaved fragments of caspase 8 and caspase 9, respectively. Cells that were not treated with p-PD (0) but with 0.1% DMSO served as controls. (b) Cells treated for 48 hours were lysed and analysed by western blot as mentioned above by probing with antibodies against active caspase 9 and Bid. “⧫” indicates both cleaved fragments of caspase 9. *β*-actin is used as a loading control for both panels. These blots are representative of three independent analyses.

In apoptotic cells, the activation of PARP could be the result of two divergent pathways, extrinsic and intrinsic, for which activation of caspases 8 and 9 is the respective signature [34, 35]. To delineate the possibilities, we have used the same cell lysates that were prepared to show the activation of PARP (as in Figure 3) to check the presence of the active forms of these caspases using immunoblot. Interestingly, in the shorter time points (6 and 24 hours), only caspase 8 gets activated (Figure 5(a), lanes 3, 4, and 6–8). Activation of caspase 9 is only seen in the samples treated for 48 hours (Figure 5(b), lanes 2–4). It is known that activated caspase 8 may lead to activation of Bid, a Bcl-2 family protein which in turn destabilizes mitochondrial membrane to initiate caspase 9 mediated intrinsic pathways [35]. To explore this possibility, we have checked the decrease of full-length Bid in the cell lysates using western blot with anti-Bid antibody. At 6 and 24 hours' time points, the amount of Bid remained unaltered (Figure 5(a), lanes 1–8), but its gradual decrease is observed in samples treated with increasing concentration of p-PD for 48 hours (Figure 5(b), lanes 1–4). It is apparent from these results that the extrinsic pathway is activated before the intrinsic one in p-PD treated melanoma cells.

### 3.4. p-PD Mediated Apoptosis of Melanoma Cells Requires Early Activation of Caspase 8

**Figure 6:**
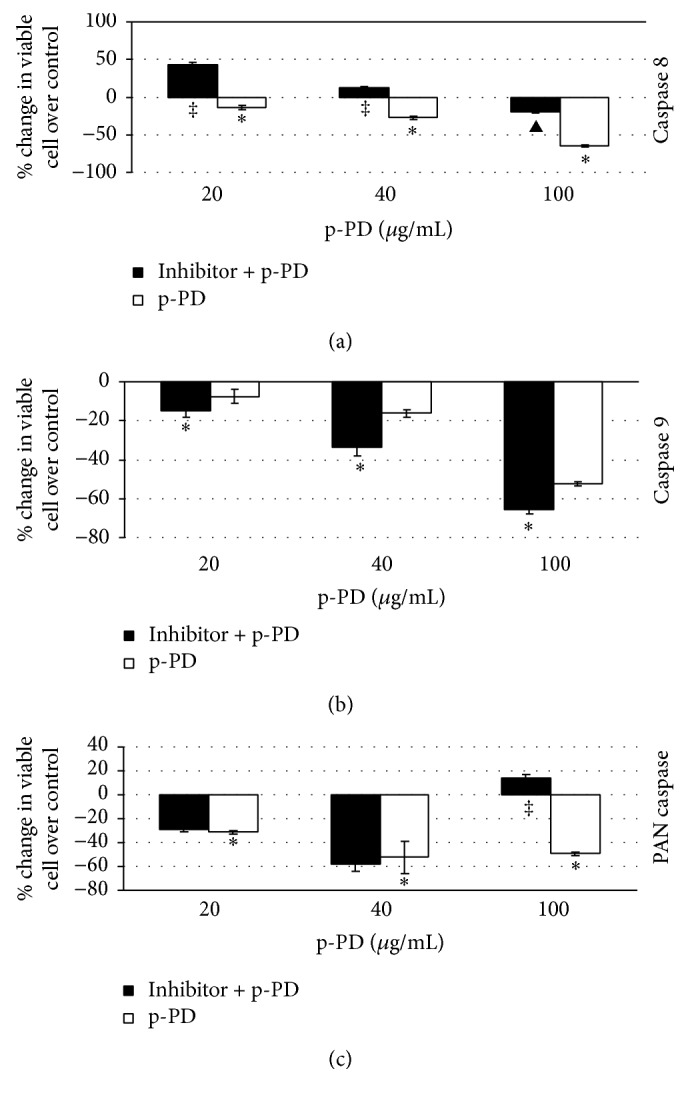
Caspase 8 inhibitor decreases the number of dead A375 cells. Cells were pretreated with the inhibitors of caspase 8 (a), caspase 9 (b), and PAN caspase (c) for two hours and subsequently treated with p-PD for 24 hours. Percent change in viable cells in sets with inhibitor pretreatment was computed as described in the Materials and Methods. The net protective effect (‡) is either the |value of white bar + value of black bar| or the decrease in dead cell number as denoted by ▲. By conventional criteria, this difference is considered to be extremely statistically significant. *∗* indicates the two-tailed *p* value ≤ 0.005. Each set was performed in triplicate.

Pharmacological inhibitors of caspases are used to identify the primary caspase, the activation of which is responsible for the initiation of apoptosis [36]. We have performed experiments using different caspase inhibitors and assessed cell viability and apoptosis in response to p-PD treatment. For that, cells that were incubated with caspase inhibitors were treated with p-PD for 24 hours and cell viability was measured by MTT based assay. The result clearly demonstrated that cells pretreated with caspase 8 inhibitor showed significant increase in viable cell (‡, Figure 6) or decrease in dead cell numbers (▲, Figure 6) upon p-PD treatment as compared to cells without the inhibitor (Figure 6). The protective effects of the inhibitor of caspase 8 were 55, 41, and 45% for 20, 40, and 100 *μ*g/mL of p-PD, respectively. It is obvious that this effect of the inhibitor is more in the cells treated with lesser p-PD. While the inhibitor of caspase 9 showed no protection in p-PD induced cell death, the PAN inhibitor of caspases significantly prevented cell death induced by higher dosage of p-PD in particular.

This protective effect of caspase 8 inhibitor on apoptosis was also verified by flow cytometry experiments where the numbers of Annexin V positive cells in response to p-PD (24 hours) were reduced by 37 and 73% for early and late apoptosis, respectively, when pretreated with caspase 8 inhibitor as compared to the “no inhibitor” set. These results demonstrate that a large extent of p-PD induced apoptotic death of melanoma cells is mediated by the activation of caspase 8.

### 3.5. Disruption of the Mitochondrial Membrane Potential

**Figure 7:**
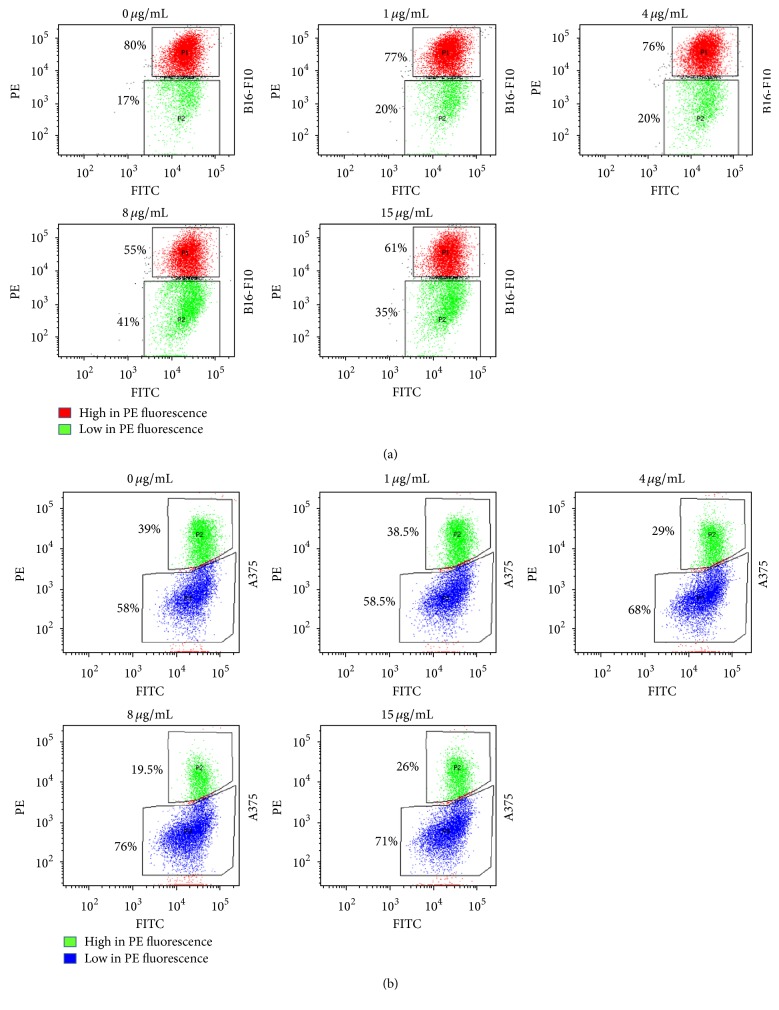
p-PD depolarizes mitochondrial membrane potential of both melanoma cells. p-PD treated (as indicated for two hours) and untreated (0 *μ*g/mL) melanoma cells were subjected to JC 1 staining for the measurement of MMP. For both cell lines, cell clusters which are high in PE fluorescence are gated in the top of each dot plot (red and green for B16-F10 and A375 cells, resp.) presenting the healthy cell population. Cell clusters that are gated for low PE fluorescence in the bottom part of each dot plot (green and blue for B16-F10 and A375 cells, resp.) are representing the cells with depolarized mitochondria. Cell percentage values of the gated population are stating the effect of p-PD in the polarization of mitochondria. One piece of the representative experimental data is shown here. Each set was performed in triplicate.

As shown above, p-PD treatment for longer time led to formation of tBid in these cells. tBid is a known destabilizer of mitochondrial membrane potential (MMP). To test whether the MMP in the treated cells have gone through any change, flow cytometric measurements were done using JC 1, a ratiometric fluorescent dye. As opposed to the result showing tBid formation, our data shows that the cells treated with 1 *μ*g/mL of p-PD for as low as 2 hours have undergone decrease in MMP (Figure 7). It is evident that, in response to p-PD treatment, the MMP is much more susceptible than the activation of either caspase 8 or Bid and thus it is presumably a direct consequence of the treatment.

### 3.6. ROS Generation upon p-PD Treatment

**Figure 8:**
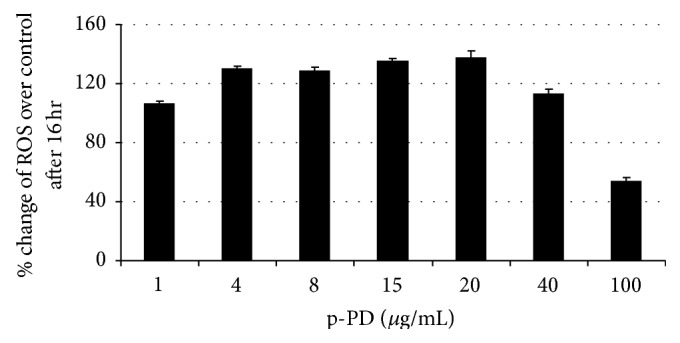
p-PD treatment increases the level of ROS in A375 cells. Control cells and cells treated with p-PD for 16 hours were subjected to DCFH-DA stain. Flow cytometric analyses of the stained cells were done as described in the Materials and Methods. One experimental data set is shown here. The percentage change of ROS was calculated using the expression (ROS_Experiment_ − ROS_Control_) × 100/ROS_Control_. Each set was performed in triplicate.

It has been demonstrated that the disruption of MMP causes ROS generation [37–39]. DCFH-DA staining was used to uncover the status of ROS in the control and treated cells. Our flow cytometry results show that up to 6 hours of p-PD treatment the ROS generation in both cell types was negligible (data not shown). It is clear that A375 cells showed significant increase in the amount of ROS after 16 hours of treatment (Figure 8). This trend continued in 24-hour treated samples (data not shown). Cells isolated from p-PD treated mice tumours also showed a substantial increase in the ROS level when compared to the untreated tumour (data not shown).

Biochemical experiments were also used to authenticate the findings. Reduction in the ratio of reduced and oxidized glutathione (GSH : GSSG) and increase in the activities of enzymes such as glutathione reductase (GR) and catalase are commonly used as standard indicators for cellular oxidative stress/ROS. The result of our measurements demonstrates that the catalase and GR activities were increased by 22 ± 2% and 39 ± 8.6%, respectively, in the lysates of cells treated with 100 *μ*g/mL of p-PD for 24 hours (Table 4). There was a substantial reduction in the amount of GSH (70 ± 13%) resulting in an 80 ± 9% decrease of GSH : GSSG ratio in the p-PD treated cells. All these biochemical lines of evidence in conjunction with the flow cytometric data strongly indicate that p-PD treatment actually imparts a strong oxidative stress to the cells.

At this point, we have used NAC and glutathione (GSH) pretreatment before p-PD exposure to validate the effect of ROS on apoptosis of melanoma cells. It is clear from Table 5 that these pretreatments, specifically NAC, decreased the amount of ROS induced by p-PD (40 *μ*g/mL). These pretreatments also reduced extent of apoptosis in p-PD treated cells (Table 5). This result strongly established a correlation between the ROS generation and the apoptotic death of p-PD treated melanoma cells. Furthermore, to investigate whether p-PD mediated depletion of MMP is dependent on ROS or not, a similar experiment with NAC was done. Flow cytometry analysis using JC 1 confirms that MMP destabilization is independent of ROS generation indicating that ROS production is downstream to the disruption of MMP.

However, the increase in ROS in response to p-PD could not be obliterated by blocking the activation of caspase 8 (data not shown) suggesting the fact that stimulation of ROS in p-PD treated cells occurs independently of the activation of caspase 8.

Although the p-PD induced apoptotic death of melanoma cells is concurrent with initial loss of MMP, activation of caspase 8, and generation of ROS, inhibition of caspase 8 activation did not have any effect on the ROS production, the sequestration of which by NAC also could not disrupt MMP. However, suppression of ROS by NAC showed marked reduction in p-PD mediated apoptotic death* in vitro* (Table 5). This observation in conjunction with the fact that the loss of MMP occurred much earlier than the induction of ROS clearly indicates that the mitochondria are one of the primary targets of p-PD (see text). The activation of caspase 8 which took place after the initiation of loss in MMP but before the generation of ROS suggests that mitochondria possibly have a role in its activation as shown elsewhere [40]. It seems that the activation of caspase 8 and the stimulation of ROS take place via two independent pathways that may subsequently have positive feedback on each other. Indeed, there are reports that showed similar interactions between ROS and activation of caspase 8 [41, 42]. Although disruption of MMP occurred very early in response to lower concentration of p-PD, the activation of caspase 9 was only observed in cells treated with higher concentration of p-PD for 24 hours. This can be explained by citing examples of previous reports which state that loss in MMP especially by small reducing substrates does not necessarily lead to the release of cytochrome C [43, 44]. In our case, p-PD being a cell permeable reducing agent may have caused the disruption of MMP without any appreciable release of cytochrome C as evident from no activation of caspase 9 at the same time.

## 4. Conclusion

Skin cancer is the most dreadful malignant tumour by virtue of its strong resistance to known chemotherapy. The incidence of skin cancer is on the rise especially among the Caucasian population [2, 3]. Case fatality for melanoma is probably the highest, not only among skin cancers but also among all types of cancers [45, 46]. At present, high-dose interleukin-2 (IL-2) and more recently anti-CTLA-4 (Cytotoxic T-Lymphocyte Antigen 4) that works through activation of the immune system are the only therapies resulting in long-term disease-free intervals [47]. Despite decades of systematic therapeutic investigations using cytotoxic, immunologic, and now molecularly targeted agents, the survival rate among treated patients with advanced melanoma is very discouraging [48]. Our study shows for the first time that both human and mouse melanoma cells undergo ROS mediated apoptotic death when treated with p-PD but not with o-PD* in vitro*. Most importantly, peritoneal injection of 2 and 4 mg/kg of p-PD inhibited the growth rate of B16-F10 induced melanoma tumour in mice (Figure 2). However, month-long peritoneal administration of as high as 100 mg/kg of p-PD did not show any sign of toxicity in Swiss-Albino mice. The total number of Annexin V positive cells is increased in tumours of p-PD treated mice. This observation substantiates our* in vitro* finding that p-PD mediates apoptotic death of melanoma cells.

Taken together, our observations suggest that p-PD mediates apoptotic destruction of human and mouse melanoma cells primarily via the loss of MMP, activation of caspase 8, and ROS generation. We have undertaken experiments to get a deeper insight into the mechanism of the apoptotic death of melanoma cells by p-PD and this study creates a platform to develop it as an agent that can successfully kill melanoma cells which are otherwise resistant to standard chemotherapeutics.

## Figures and Tables

**Table 1:** Effect of p-PD treatment on body weight with major organ weight in mice.

Treatment	Initial body weight (g)	Final body weight (g)	Final heart weight (g)	Final lung weight (g)	Final liver weight (g)	Final kidney weight (g)	Final spleen weight (g)
Control (vehicle only)	22 ± 0.33	25 ± 0.06	0.16 ± 0.02	0.16 ± 0.06	1.7 ± 0.04	0.35 ± 0.12	0.26 ± 0.03
Treatment dose 1^*∗*^	25 ± 0.09	29 ± 0.11	0.16 ± 0.04	0.13 ± 0.09	1.92 ± 0.03	0.38 ± 0.06	0.27 ± 0.03
Treatment dose 2^*∗*^	20 ± 0.04	24 ± 0.12	0.15 ± 0.06	0.18 ± 0.02	1.57 ± 0.08	0.32 ± 0.05	0.24 ± 0.04

^*∗*^Dose 1 and dose 2: p-PD 5 and 10 mg/kg/3 days, respectively.

Values are expressed as mean ± SEM (*n* = 10).

**Table 2:** Effect of p-PD treatment on serum biochemical parameters of mice.

Treatment	SGOT (IU/dL)	SGPT (IU/dL)	SALP (IU/dL)	Bilirubin (mg/dL)	ALT (IU/dL)	AST (IU/dL)	ALP (IU/dL)	Urea (mg/dL)	Uric acid (mg/dL)	Creatinine (mg/dL)
Control (vehicle only)	43 ± 11.01	35 ± 5.22	84 ± 8.33	1.0 ± 0.12	46 ± 5.23	95 ± 14.33	286 ± 7.36	155 ± 9.36	7 ± 9.33	1.0 ± 1.22
Treatment dose 1^*∗*^	41 ± 6.02	34 ± 4.12	89 ± 3.63	1.0 ± 1.32	47 ± 6.03	97 ± 16.33	296 ± 9.36	157 ± 11.23	7 ± 14.36	1.0 ± 1.88
Treatment dose 2^*∗*^	45 ± 6.14	37 ± 9.11	86 ± 9.32	1.0 ± 2.33	43 ± 9.54	92 ± 3.63	289 ± 9.36	165 ± 18.33	6 ± 9.36	1.0 ± 1.22

^*∗*^Dose 1 and dose 2: p-PD 5 and 10 mg/kg/3 days, respectively.

Values are expressed as mean ± SEM (*n* = 10).

**Table 3:** Quantification of apoptosis.

Cell status	Control	24 hours			Control	48 hours		
		p-PD (*µ*g/mL)				p-PD (*µ*g/mL)		
		20	40	100		20	40	100
	A375							
Live^a^	92 ± 1	90 ± 1.3	89 ± 4.34	81 ± 0.34	91 ± 0.85	59 ± 1.3	42 ± 0.23	36 ± 0.6
Early apoptosis^a^	3 ± 0.2	3 ± 0.5	5 ± 0.3	11 ± 0.19	6 ± 0.93	11 ± 0.4	17 ± 0.2	21 ± 0.58
Late apoptosis^a^	4 ± 0.4	6 ± 0.4	6 ± 0.3	7 ± 0.43	2 ± 0.1	27 ± 0.3	39 ± 0.5	40 ± 0.86
Sub-G0/G1^b^	10 ± 1.8	11 ± 0.5	15 ± 2.2	24 ± 4	10 ± 2.8	38 ± 6.4	39 ± 5.1	50 ± 7.3

	B16-F10							
Live^a^	87 ± 6.8	75 ± 7.4	73 ± 6.6	71 ± 6.8	92 ± 4.1	44 ± 5.3	36 ± 2.4	8 ± 1
Early apoptosis^a^	6 ± 0.3	16 ± 3.2	17 ± 3.4	17 ± 2.2	4 ± 0.8	32 ± 4.1	36 ± 3.1	42 ± 1.9
Late apoptosis^a^	5 ± 0.8	7 ± 2	9 ± 3	11 ± 1.6	4 ± 0.6	21 ± 1.9	23 ± 2.5	43 ± 1.8
Sub-G0/G1^b^	1 ± 1.5	4 ± 1	6 ± 1.4	10 ± 1.2	2 ± 0.1	22 ± 3.1	55 ± 4.4	60 ± 2.9

^a^The percent cell numbers of early and late apoptotic stages were determined from the respective dot plots (one such plot is shown in Figure 3(b)).

^b^The sub-G0/G1 cells were estimated using standard cell cycle analysis by flow cytometry.

All the sets were performed in triplicate.

p-PD treated and untreated (control) A375 andB16-F10 cells were subjected to Annexin V-FITC assay as mentioned in the Materials and Methods.

**Table 4:** Change in the biochemical parameters of oxidative stress induced by 100 *µ*g/mL of p-PD for 24 hours on A375 cells.

Glutathione reductase			Catalase			GSH			GSH : GSSG		
p-PD (*µ*g/mL)		% change	p-PD (*µ*g/mL)		% change	p-PD (*µ*g/mL)		% change^a^	p-PD (*µ*g/mL)		% change^a^
Cont.	100		Cont.	100		Cont.	100		Cont.	100	
12 ± 1.5	17 ± 0.5	39 ± 8.6	5 ± 0.3	6 ± 0.2	22 ± 2	19.2 ± 0.7	5.9 ± 1.9	70 ± 13	62 ± 26	12 ± 4	80 ± 9

^a^These changes indicate percent decrease of the respective parameters in the treated samples. Each set of data is averaged from three different experiments.

Oxidative stress was measured using the standard parameters such as reduced GSH amount, GSH : GSSG, and activity of glutathione reductase and catalase. Parameters were measured as described in Section 2.5. We used control cells and cells treated for 24 hours with 100 *µ*g/mL of p-PD for these experiments. GSH was expressed as nmol mg^−1^ protein; catalase and glutathione reductase activities were expressed as nmol min^−1^ ****mg^−1^ protein.

**Table 5:** Effect of NAC and GSH pretreatment on ROS generation and apoptosis in A375 cells.

p-PD (*µ*g/mL)	% decrease in ROS		% drop in apoptosis	
	NAC (10 mM) pretreatment	GSH (5 mM) pretreatment	NAC (10 mM) pretreatment	GSH (5 mM) pretreatment
0	34 ± 3.5	−6 ± 0.9	0	0
20	31 ± 2.6	34 ± 1.4	63 ± 4.7	41 ± 5.6
40	66 ± 4.5	29 ± 2	47 ± 4	18 ± 0.2
100	25 ± 1.2	6 ± 0.3	41 ± 2.1	44 ± 3

A375 cells were divided into 2 groups: one was pretreated with NAC or GSH for 1 hour and then treated with p-PD for 16 hours and the other was treated with only p-PD. ROS values and apoptotic cell numbers were quantified at the end of p-PD exposure as mentioned in the Materials and Methods. Percentage changes in ROS and apoptosis were calculated as follows: % change = {(only p-PD value − respective pretreatment value)/respective pretreatment value}  ×  100. Values are representative of three independent experiments.
